# Algorithm-Guided Management of Thumb Amputation: A 20-Year Retrospective Review and Outcome Analysis

**DOI:** 10.3390/jcm14228250

**Published:** 2025-11-20

**Authors:** Maja Smorąg, Piotr Węgrzyn, Marta Jagosz, Michał Chęciński, Szymon Manasterski, Jędrzej Króliński, Marcin Syrko, Patryk Ostrowski, Katarzyna Kościelska-Kasprzak, Dorota Kamińska, Ahmed Elsaftawy

**Affiliations:** 1 Department of Plastic and Hand Surgery, St. Jadwiga Śląska Hospital, 55-100 Trzebnica, Poland; maja.smorag@gmail.com (M.S.); wengrzu@gmail.com (P.W.); marta.malgorzata.jagosz@gmail.com (M.J.); michal.checinski@gmail.com (M.C.); smanasterski@gmail.com (S.M.); jedrzej.krolinski@gmail.com (J.K.); marcinsyrko@gmail.com (M.S.); elsaftawyahmed@gmail.com (A.E.); 2 Department of Anatomy, Jagiellonian University Medical College, 31-008 Kraków, Poland; ostrowskipatryk0@gmail.com; 3 Faculty of Medicine, Wroclaw University of Science and Technology, 50-370 Wroclaw, Poland; d.kaminska@pwr.edu.pl

**Keywords:** traumatic thumb amputation, replantation

## Abstract

**Background:** The thumb accounts for 40–50% of hand function. Traumatic amputation of the thumb results in significant disability and necessitates a structured approach to management. **Methods:** We conducted a retrospective review of 144 patients treated for thumb amputations between 2004 and 2025 at a specialist hand surgery unit. Over 21 years, an institutional algorithm was developed and refined to guide treatment decisions based on amputation level, injury mechanism and patient factors. **Results:** Out of the 144 cases, 118 patients underwent replantation, achieving an 82% success rate. Alternative reconstructive procedures included toe-to-thumb transfers (eight cases), index finger pollicisation (six cases) and fourth finger pollicisation (five cases). Functional outcomes showed that 90% of patients returned to work, 83% regained temperature and touch sensation, and 94% could lift a 0.5 L bottle. **Conclusions:** Implementing a structured treatment algorithm facilitates personalised care and leads to favourable functional outcomes in patients with traumatic thumb amputations.

## 1. Introduction

The thumb plays a critical role in hand function, accounting for approximately 40–50% of overall hand utility. Its unique anatomical position and biomechanical design enable opposition, grip strength, fine manipulation and sensory feedback, making it essential for performing both basic and complex tasks [1]. Traumatic thumb amputations, which are often the result of guillotine, crush, or avulsion injuries, represent a significant loss of function with far-reaching consequences for patients’ quality of life, occupational capacity, and psychological well-being.

The primary objective of the initial management of thumb amputation is to salvage and restore the original anatomy through microsurgical replantation, provided it is technically feasible. Replantation offers the best potential for regaining sensitivity, stability, and dynamic function [2,3,4]. However, not all cases are suitable candidates for replantation. The mechanism of injury, ischaemia time, the condition of the amputated part and patient-specific factors such as comorbidities or occupational demands all influence the choice of treatment.

We developed an institutional algorithm to guide management decisions based on the level of amputation, injury mechanism and patient-specific factors. Over the course of 21 years, we have refined an institutional algorithm that addresses both acute and delayed cases of thumb amputation. This algorithm encompasses a full spectrum of interventions, from replantation to complex reconstructions and prosthetic rehabilitation, allowing for evidence-based, individualised treatment decisions. This study aims to present the outcomes of our long-term experience and provide a structured, pragmatic approach to the surgical management of traumatic thumb amputations in different clinical scenarios.

## 2. Materials and Methods

### Patient Selection and Data Collection

The study included a retrospective cohort of 144 patients who were treated for thumb amputation in Hand Trauma Center of St. Jadwiga Śląska Hospital, Trzebnica, Poland, between 2004 and 2025. All procedures were performed by surgeons with subspecialty training in hand surgery in accordance with the World Medical Association Declaration of Helsinki.

During this period, the patients were treated for thumb amputations with the following procedures: replantation (118 cases), toe-to-thumb transfer (8 cases), index finger pollicization (6 cases), the fourth finger pollicization (4 cases), radial forearm flap (3 cases), McGregor flap (2 cases), and Foucher flap (3 cases).

The data collected for the purpose of the study included patient demographics, the mechanism of injury, the level of amputation, the treatment modality and the functional outcomes. The primary measures of achievement are anatomical survival of the replanted thumb and restoration of useful pinch or opposition function. These are referred to as success and functional recovery, respectively. Due to incomplete data for the earlier period, the available performance data is presented in descriptive form, and no in-depth statistical analysis was applied.

All clinical photographs were used with written informed consent from the patients involved, and all images were anonymized in accordance with institutional ethical standards. The present study was conducted using retrospective, anonymized medical data, and as such, did not require approval from an ethics committee.

## 3. Results

### 3.1. Replantation

**Figure 1:**
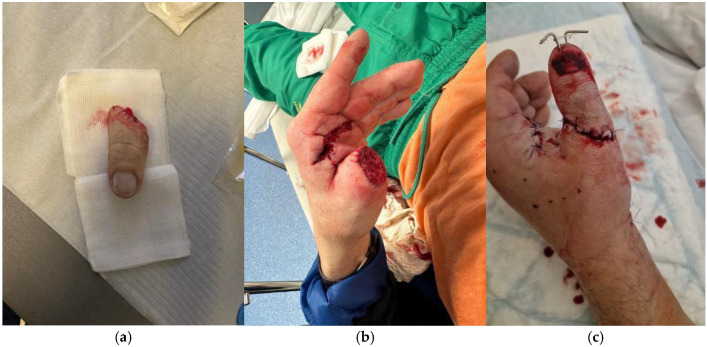
A case of a 51-year-old patient, manual worker who presented with thumb amputation caused by a circular saw. (**a**) Amputated part of the thumb. (**b**) The stump with a sharp cut edge; (**c**) Presentation of the thumb after the replantation procedure.

**Figure 2:**
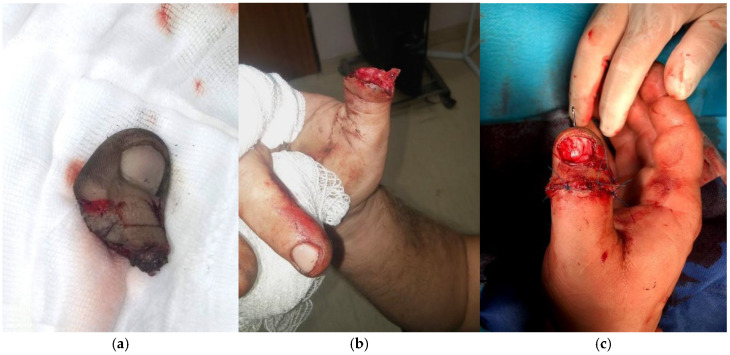
A case of a 42-year-old manual worker who had an amputation of the thumb at the interphalangeal joint caused by an axe. (**a**) The amputated right thumb; (**b**) The stump; (**c**) The replanted thumb showing a good blood supply after surgery.

During this period, we successfully performed 118 thumb replantations on predominantly male patients with a median age of 47 years (range 16–87 years). Hospital stays ranged from three to 25 days, with an average of 10 days. The majority of injuries were caused by a circular saw (42%) (Figure 1). The remaining injuries were caused by axe cuts (Figure 2), angle grinders and crushing by a stone crusher. Complications, including skin necrosis and joint stiffness, were documented.

Replantation procedures involved debridement, skeletal fixation using Kirschner wires, tendon repair, vascular anastomosis, nerve approximation, and soft tissue closure. Postoperative care included anticoagulation therapy with therapeutic doses of heparin for 5 days after the surgery, infection control, immobilization, elevation of the limb and intensive rehabilitation.

Rehabilitation commenced immediately after surgery. Passive and active exercises, as well as sensory re-education, were introduced progressively over a 12-week period to restore range of motion, strength, and sensory function.

Due to difficulties in obtaining complete documentation of patients from earlier years, we collected functional results of patients operated on during the period 2019 to 2024. During this time, we performed 22 thumb replantations. Ninety per cent of patients returned to work, with four out of five of those who had not resumed employment having undergone surgery within the previous six months. Thirty-three per cent of patients performed manual labour. Based on patients’ self-report, sensory recovery was noted in 83% of patients for both temperature and touch sensation. Pain, as assessed by VAS, averaged 2/10. Functionally, 93% of patients could lift a 0.5 L bottle and 73% could lift a 1.5 L bottle. Joint stiffness and limited range of motion were observed in 50% of cases, with a Kapandji test score of 5 or lower indicating ongoing difficulties. Among patients with failed replantation, the mechanisms of injury included one case of avulsion, one clean cut caused by a saw, and two crush injuries.

We observed a number of factors that influenced the outcome for each of the patients: mechanism of injury, ischemia time, patient factors, level of amputation, availability of the amputated part, and postoperative care. A more detailed description of the factors is presented in Table 1.

### 3.2. Reconstruction Using Flap Coverage

If replantation is not possible or if complications arise during thumb replantation, such as necrosis, soft tissue loss or tendon exposure, before the appearance of mummification symptoms, local and regional flaps play a critical role in salvage.

**Figure 3:**
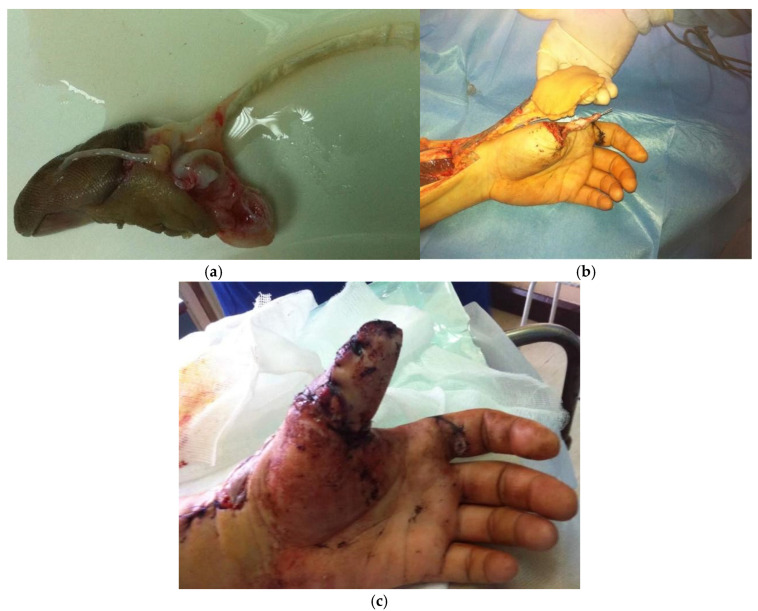
A case of a 37-year-old patient who underwent avulsion amputation of the thumb. As replantation was not possible due to extensive tissue damage, the decision was made to skeletonise the amputated phalanges and cover them with a pedicled radial forearm flap. (**a**) Amputated thumb; (**b**) Appearance of the hand immediately prior to covering the defect with a pedicled radial forearm flap following thumb skeletonisation with K-wire fixation; (**c**) Postoperative result of the thumb one week after the procedure, demonstrating satisfactory healing and coverage.

We start with the procedure of necrectomy or skeletonization of the amputated thumb. Then the choice of flap is determined by the extent and location of the defect. The radial forearm flap (Figure 3) offers a well-vascularized, pliable solution for larger soft tissue deficits, particularly when durable coverage is needed over joint structures. The Foucher flap, based on the first dorsal metacarpal artery, provides sensate coverage and is particularly suited for smaller distal defects. Wang et al. also described the use of a reverse homodigital dorsal wraparound flap for distal thumb reconstruction [5].

**Figure 4:**
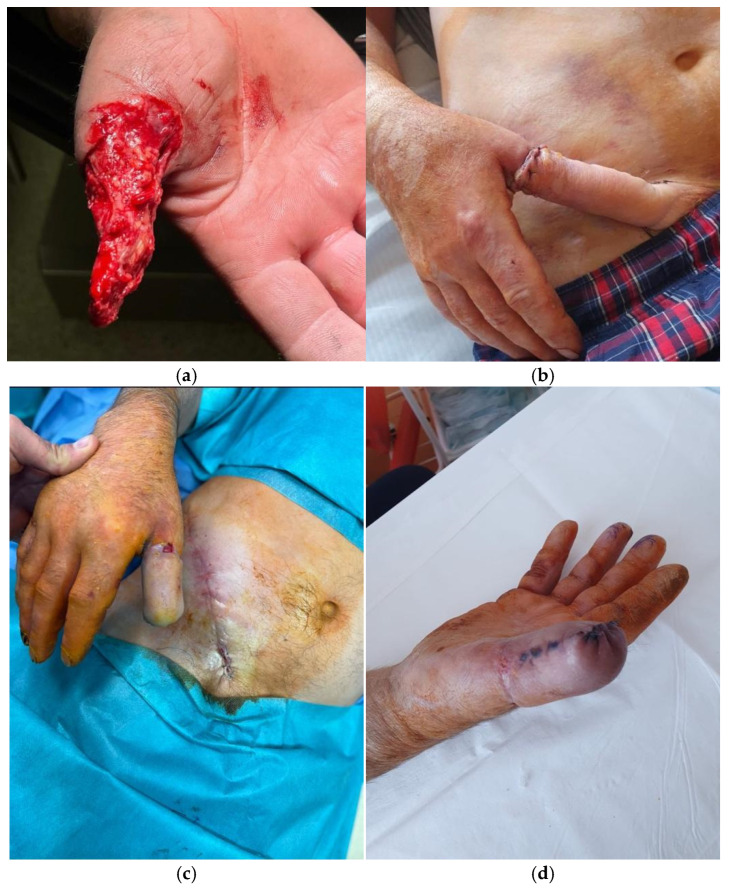
A case of a 62-year-old patient with an avulsion injury to the thumb above the metacarpophalangeal joint. The tissue defect in the thumb was covered with a pedicled inguinal flap. (**a**) Skeletonised thumb with preserved tendons and neurovascular bundles; (**b**) Inset of the pedicled inguinal flap onto the defect; (**c**) Result after flap division, three weeks after the first stage; (**d**) Result showing satisfactory healing and coverage, fourteen days postoperatively.

In avulsion-type amputations, where extensive soft tissue destruction often renders replantation infeasible or predictably unsuccessful, we advocate for an alternative approach using the pedicled McGregor flap (Figure 4). While this technique is rarely reported in recent literature and sometimes perceived as outdated, our experience has demonstrated its practical advantages. When performed urgently, the McGregor flap enables rapid coverage of the residual thumb structure, preserving fundamental grasp function and facilitating early rehabilitation. In selected cases, it remains a valuable and effective solution, especially when microsurgical resources are limited or delayed. This kind of procedure preserves native bony structure, avoids complete amputation and prosthetic dependence and maintains proprioception and some tactile sensation. As a secondary procedure in the future, an innervated island as in Littler flap can be carried out [6].

Unfortunately, the disadvantages of these procedures are multiple surgeries required, donor site morbidity and limited aesthetic results compared to full thumb reconstruction.

In our centre we have performed three radial forearm flap procedures for stump coverage, three Foucher flap procedures and in two patients, the defect was treated using an inguinal flap, all of them with good functional and aesthetic results (Kapandji opposition score: median 6, range 4–9; range of motion of the reconstructed thumb: mean MCP arc 42° ± 8°, IP arc 28° ± 6°; grip and pinch strength measured with Jamar dynamometer: key pinch mean 63% and tip pinch mean 58% of the contralateral side). In the patients mentioned, an initial attempt at replantation was performed. Subsequently, after the development of necrosis in the soft tissues, the necessity for revision surgery was determined. The revision procedure was carried out between 4 and 15 days following replantation. The choice of flap type was made based on the anatomical characteristics of each patient.

### 3.3. Toe-to-Thumb Transfer

For complete thumb amputations, particularly at the MP joint or proximal level, toe transfer remains a well-established reconstructive option. This operation is performed in a delayed manner. The first microsurgical great toe-to-thumb transfer in humans was carried out by Cobbett in 1969. This was soon followed by the transfer of a second toe to the thumb position. Depending on the level of injury and the extent of tissue loss, this technique allows for consideration of either a total second toe transfer or a transfer including the metacarpal bone, which enables reconstruction of the entire first radius. This procedure restores functional opposition and grip, provides superior sensory feedback compared to prosthetics or pedicled flaps, and eliminates the need for additional interventions. Furthermore, it offers a more favourable aesthetic integration. However, there are several disadvantages, including donor site morbidity, the complexity of the surgical procedure, and the potential risk of partial or complete failure due to vascular complications.

**Figure 5:**
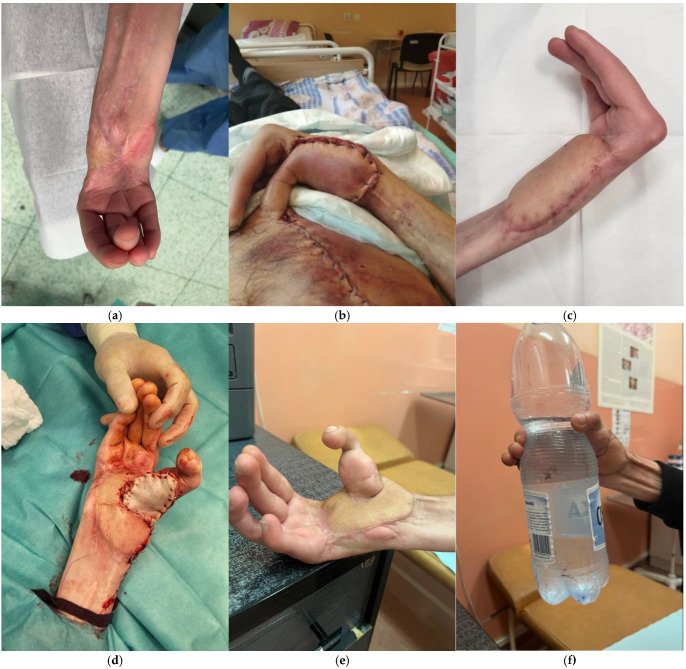
A case of a 44-year-old patient who had undergone amputation of the thumb, including the first metacarpal bone and the thenar muscles. The patient had a postoperative scar at the site of the thumb defect and experienced stiffness in the interphalangeal joints. The patient was admitted to our hospital three years after the injury. It was decided that the second toe, together with the metatarsal bone, would be transferred from the foot to recreate the first ray of the hand and the first webspace. The lack of the TMC joint meant that he was unable to hold a precise grip or key pinch; however, he was able to grip larger objects. Prior to the procedure, the entire scar on the hand was removed and the defect was covered with a pedicled inguinal flap. The procedure to transfer the second toe took place six months after the inguinal flap was detached. (**a**) Initial presentation of the patient’s hand on admission, before scar removal; (**b**) Inset showing the pedicled abdominal flap covering the defect after scar removal; (**c**) Result after the first stage of treatment; (**d**) Appearance of the hand after transfer of the second toe and second metatarsal bone; (**e**) Final presentation of the hand, demonstrating satisfactory restoration, 10 months postoperatively, (**f**) Functional result.

In eight cases of second-toe transfer to the thumb position, the average MCP arc measured 41° ± 8°. All patients regained precision pinch function. Functional outcomes assessed by the Percival classification showed five excellent and three good results. In all operated cases, functional hand grasp was successfully restored. Postoperative rehabilitation played a crucial role in optimizing functional recovery, focusing on both range of motion and sensory re-education. No major donor site complications were observed, and all patients reported satisfactory aesthetic and functional results. These encouraging outcomes reinforce the reliability of the second toe transfer as a valuable reconstructive option for thumb amputations, particularly when preserving hand functionality and sensation are paramount (Figure 5).

### 3.4. Utilization of Other Amputated Fingers (In Whole or in Part) for Thumb Reconstruction

**Figure 6:**
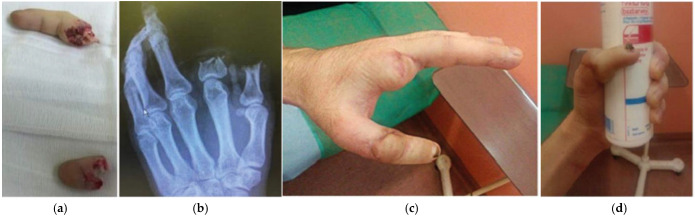
A case of a 24-year-old patient who had their thumb and index finger amputated. The thumb was not suitable for replantation due to the nature of the injury. It was therefore decided to replant the index finger in place of the lost thumb. (**a**) Presentation of the amputated fingers: the index finger (upper part of the figure) and the amputated thumb (lower part); (**b**) X-ray taken after the injury; (**c**,**d**) Final appearance after surgery, showing functional and aesthetic restoration five months postoperatively.

In case when several fingers are amputated along with the thumb, when the thumb amputate is not suitable for replantation, another amputated finger can be used in part or in whole to reconstruct the thumb. As in the previous case, the main disadvantages of the procedure are the complexity of the surgical procedure, and the risk of partial or complete failure due to vascular complications. Additionally, the success of the procedure also depends on the condition of the replanted finger. In our material it most often concerned amputation of the index finger alongside amputation of the thumb. The presented case from our center (Figure 6) illustrates a 24-year-old patient with traumatic amputation of both the thumb and index finger, where the thumb was deemed unsuitable for replantation due to the injury pattern. The index finger was successfully replanted in the thumb position, resulting in satisfactory functional and aesthetic outcomes, as demonstrated by the postoperative images five months after surgery.

**Figure 7:**
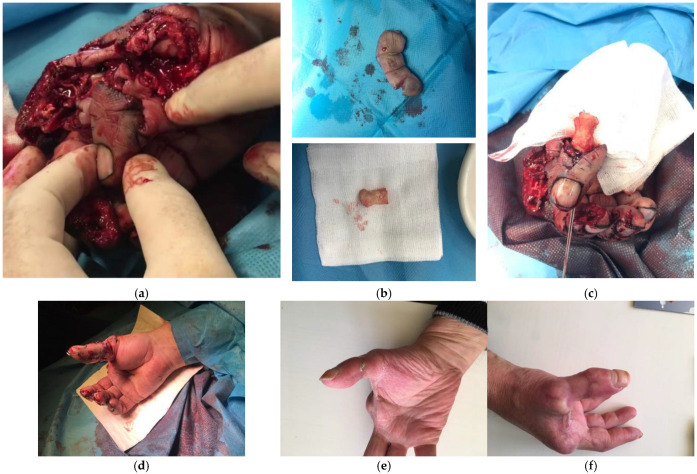
This is a case study of a 47-year-old patient who had undergone amputation of the thumb and index finger. The thumb was missing its proximal phalanx, so the amputated index finger was used to restore thumb function. The middle phalanx of the index finger was skeletonised and placed in the place of the lost proximal phalanx of the thumb to reconstruct the height and function of the thumb. (**a**) Initial presentation of thumb injury; (**b**) parts of index finger: proximal phalanx (below) and soft tissue residue (above); (**c**) connection of proximal index phalanx with amputated thumb before replantation procedure; (**d**) postoperative appearance; (**e**,**f**) final presentation 6 months postoperatively showing good aesthetic and functional results.

The second case operated in our department was a 47-year-old patient with traumatic amputation of both the thumb and index finger. Notably, the thumb amputation included loss of the proximal phalanx. To restore thumb function, the amputated index finger was utilized as a donor. The middle phalanx of the index finger was skeletonized and transplanted in place of the missing proximal phalanx of the thumb to reconstruct both the length and functionality of the thumb. The final outcomes at six months demonstrate satisfactory aesthetic integration and restoration of functional hand use (Figure 7).

### 3.5. Index/4th Finger Pollicization

Due to the possibilities offered by flap reconstructions and toe-to-hand transfer, the method of thumb pollicization has seen a decrease in its application. Originally developed for cases of congenital thumb absence, this technique has also been successfully adapted for use in post-traumatic cases, achieving favourable outcomes in terms of strength and function [7,8]. It remains a good solution in cases of thumb amputation with damage to the thenar muscles. This procedure should be performed in a delayed fashion, after complete tissue healing. Vascular damage, as well as complete or partial tissue necrosis, may occur; however, with careful and planned execution of the procedure, these complications are not frequent.

**Figure 8:**
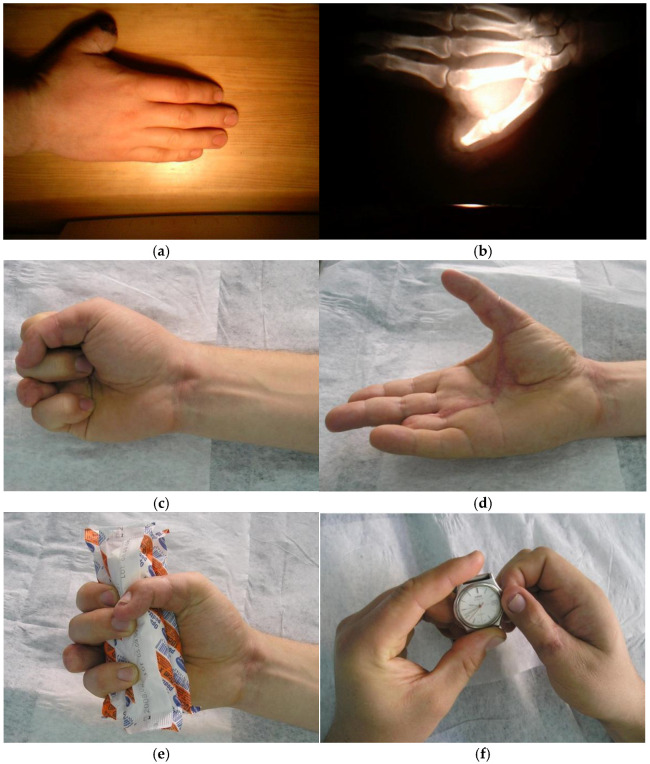
A case of a 25-year-old male manual worker who was admitted for thumb function reconstruction after amputation and treatment at another centre. The patient was offered pollicisation of the distal part of the fourth finger at the level of the proximal interphalangeal joint. (**a**) Initial presentation of the thumb, three months after amputation; (**b**) X-ray of the hand on arrival at our centre; (**c**,**d**) Final presentation showing a satisfactory aesthetic outcome after the procedure, with the thumb flexed and abducted; (**e**,**f**) Functional result showing good grasp and key pinch of the operated limb six months after the described surgical treatment.

In cases where the thenar muscles are preserved and the patient is a manual worker, pollicization of the two distal phalanges of the fourth finger (from the level of the proximal interphalangeal joint) is an option that we always consider (Figure 8). It allows for the maintenance of the key pinch. The functional grasp of the hand remains almost identical and is superior to that observed in patients who undergo pollicization of the second finger. Aesthetic outcomes may be further improved by resection of the entire fourth radius, resulting in a four-fingered hand. For some well-selected patients, pollicisation of the distal phalanx of the ring finger may be an option [9]. For six index-finger and four ring-finger pollicizations, the mean MCP arc was 45° ± 7°. Precision pinch ability was restored in all cases. The age-adjusted functional scores, based on the Percival classification, were 98% excellent and 2% good results.

### 3.6. Prosthetic Rehabilitation for Non-Surgical Candidates

For patients who are not suitable for surgery or who decline reconstruction, prosthetic rehabilitation is an important alternative. Although modern silicone prostheses can provide an aesthetic improvement and limited functional assistance, they lack sensory feedback and often necessitate significant adaptation. Studies show that many patients discontinue use due to discomfort or dissatisfaction with functionality [10]. Nevertheless, for elderly patients, those with comorbidities, and individuals seeking minimally invasive options, prosthetics can offer psychological and practical benefits.

### 3.7. Algorithm of Management After Amputation of the Thumb

**Figure 9:**
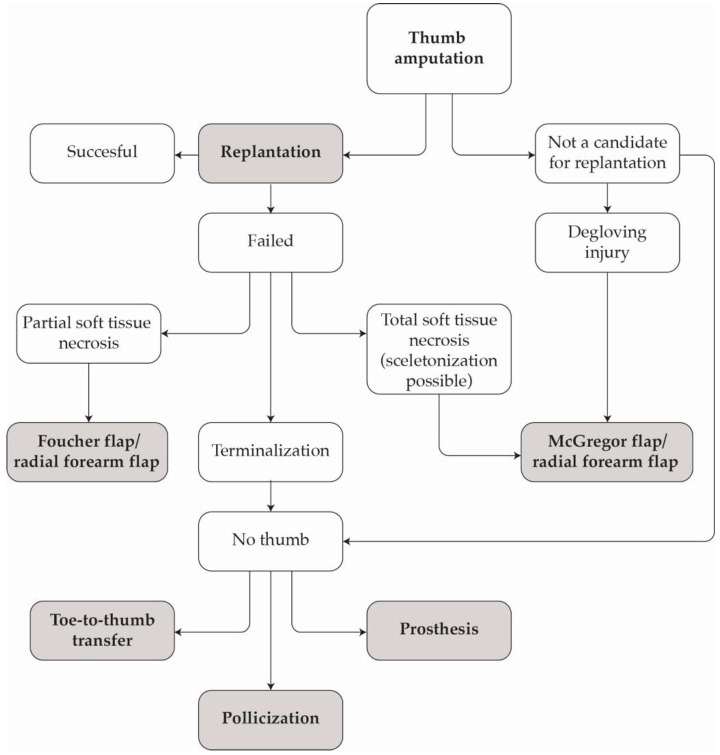
Algorithm of management after amputation of the thumb depending on the degree of damage.

Over the past two decades, our cumulative clinical experience has led to the development of a structured treatment algorithm. This is designed to guide decision-making according to the anatomical level of injury, extent of tissue viability, mechanism of trauma and patient-specific functional requirements (see Figure 9). This algorithm provides a systematic framework for evaluating each case and selecting the most appropriate reconstructive strategy within the constraints of the clinical setting.

By integrating surgical feasibility with individual patient factors, this approach has facilitated more rational treatment planning, minimised procedural variability and improved functional and aesthetic outcomes.

## 4. Discussion

Given the essential role of the thumb in opposition, grip strength, and precision handling, amputation of the thumb is one of the most functionally devastating injuries to the upper limb. Restoring thumb function is therefore a complex endeavour, requiring technical microsurgical precision and a structured, patient-specific treatment strategy.

The diversity of cases we encountered confirmed the need for such an adaptable experience-informed approach. In clinical practice, no two thumb amputations are identical, and a successful reconstruction often depends on the surgeon’s ability to apply flexible, experience-informed algorithms, rather than adhering rigidly to predetermined protocols. This adaptability ensures that technical and psychosocial factors are both adequately addressed in the overall recovery process.

Our algorithm is a comprehensive decision-making framework that integrates evidence-based surgical principles with the practical realities of personalised patient care.

Its stepwise structure enables clinicians to create reconstructive plans tailored to each patient’s anatomy and functional needs. Starting with attempts to salvage native tissue, the plans progress to more complex reconstructive options if necessary. The mechanism of injury and the time elapsed since the injury significantly impact the decision regarding replantation eligibility. Replantation should remain the gold standard, particularly for guillotine-type injuries. Crush and avulsion injuries cause more extensive soft tissue damage and therefore more often require defects to be covered with flaps. While some patients present immediately after injury, others present later, after terminalisation. At this point, they may be offered reconstructive procedures, such as toe transfer or pollicisation.

The introduction of the structured algorithm in 2018 coincided with the improved standardisation of replantation and reconstruction protocols. This has likely enhanced clinical decision-making, contributing to a reduction in complication rates. Including sensory rehabilitation and early mobilisation into the reconstructive plans further improves outcomes, as supported by an increasing body of literature on the advantages of early functional engagement following hand surgery [11,12].

Replantation remains the cornerstone of thumb reconstruction and is prioritised whenever anatomically and technically feasible. The primary advantage of replantation lies in its potential to restore the patient’s original anatomy, including vascular integrity, tendon function, and sensibility. This is particularly important in cases of clean-cut amputation with short ischaemic times. In our series, replantation success rates exceeded 80%, which is consistent with the reported survival rates in the literature ranging from 71% to 93%, depending on the injury mechanism, surgical timing and perioperative management [4,11,12,13,14,15,16,17,18,19]. Nonetheless, replantation carries inherent limitations: it requires advanced microsurgical skills, extended operating times and a narrow window for successful intervention. Furthermore, injuries involving crush or avulsion mechanisms, which we encountered in some failed cases, are associated with a higher rate of complications, including vascular compromise, tissue necrosis and ultimately, poorer functional recovery [3,4,13,14,19].

Despite continued advances in microsurgical techniques, a significant proportion of patients in our cohort either presented late or experienced early complications that prevented successful replantation. For these individuals, our algorithm emphasises the early identification of non-viable replantations and the selection of alternative reconstructive strategies, including pedicled and free flap coverage, toe-to-thumb transfers and pollicisation. These options are carefully matched to the amputation level, soft tissue condition and overall patient profile. Pedicled flaps, such as the radial forearm and Foucher flaps, have produced reliable functional and aesthetic results, reaffirming their value as salvage procedures. The selective use of less common options, such as the McGregor flap, has also been beneficial in cases where resources are limited or urgent coverage is required. Furthermore, meta-analyses have confirmed consistently high levels of long-term patient satisfaction and functional recovery following toe-to-thumb transfers, particularly in complex cases requiring complete first-ray reconstruction [20], In cases of multiple digit amputation, using another amputated finger for thumb reconstruction demonstrates the adaptability of our reconstructive approach. However, the success of such procedures depends critically on the condition of the donor tissue and its vascular viability—factors that are often compromised in high-energy or crush injuries [21].

Furthermore, a significant proportion of patients do not seek medical attention in an acute state, but seek help several weeks or months after the injury. These patients pose a particular clinical challenge. Their treatment requires a thorough assessment of their current level of fitness, professional needs, aesthetic expectations, and psychological readiness for reconstructive surgery. Some patients adapt to prosthetic solutions, while others reject them due to poor proprioception, discomfort, or professional limitations.

### Limitation of the Study

The limitations of our study include the inherent heterogeneity of the injury patterns and treatment modalities encountered over two decades. The retrospective design also limits our ability to control for confounding variables, as data were collected from existing records that may vary in terms of their completeness and accuracy. This also restricts the ability to standardize outcome assessment across all time periods. Follow-up functional results were limited to patients operated on during the period 2019 to 2024 due to difficulties in obtaining complete documentation for earlier cases. This constraint affects how generalisable the findings are and highlights the need to collect comprehensive long-term data prospectively to strengthen future analyses. Additionally, this study encompasses over twenty years of data collection, during which significant advancements have been made in microsurgical techniques in treating thumb amputation and its complications. These changes over time introduce variability in both surgical approach and outcome measurement, which could affect the comparability of results across different time frames.

## 5. Conclusions

A structured approach tailored to the patient’s specific injury and functional goals is required for the management of thumb amputation. Although replantation remains the gold standard, various reconstructive and prosthetic solutions are available when this is not possible. Although our outcomes demonstrate a high success rate and significant potential for functional recovery, challenges such as joint stiffness and incomplete sensory recovery persist. Implementing our structured treatment algorithm has led to high success rates in thumb replantation and the efficient use of reconstructive alternatives. The algorithm enables us to provide personalized care based on clinical, anatomical and functional criteria, offering clear pathways even in complex cases. Our experience supports its continued use and broader adoption.

Prospective multicentre cohort studies are planned to evaluate the algorithm’s performance over time, which would help to confirm its reproducibility and applicability to other settings. Furthermore, future research should concentrate on enhancing microsurgical techniques, promoting sensory nerve regeneration, providing personalised rehabilitation programmes and developing more advanced prosthetic solutions.

## Figures and Tables

**Table 1:** Factors influencing the outcome of thumb replantation.

Factor	Description
Mechanism of injury	Sharp, guillotine-like amputations had the highest success rates; crush or avulsion injuries lead to extensive soft tissue damage, decreasing viability
Ischemia time	Digits tolerated up to 12 h of warm ischaemia and up to 24 h of cold preservation
Patient factors	Young, healthy patients without vascular disease or severe comorbidities had better outcomes
Level of amputation	Distal amputations (at the IP joint or above) were more successful than proximal ones
Availability of the amputated part	The severed thumb must have been well-preserved for microsurgical repair
Postoperative care	Anticoagulation, infection control and intensive rehabilitation

## Data Availability

The raw data supporting the conclusions of this article will be made available by the authors on request.
